# The Challenge of Discharging Research Ethics Duties in Resource-Constrained Settings

**DOI:** 10.1371/journal.pmed.1000421

**Published:** 2011-03-15

**Authors:** Jerome Amir Singh

**Affiliations:** 1 Centre for the AIDS Programme of Research in South Africa (CAPRISA), University of Kwazulu-Natal, Durban, South Africa; 2 Dalla Lana School of Public Health, Joint Centre for Bioethics, and McLaughlin-Rotman Centre for Global Health, University of Toronto, Toronto, Canada

## Abstract

Jerome Singh discusses some ethical issues raised by a new research article by Edward Jónes-Lopez and colleagues that examined the effectiveness of the standard WHO recommended retreatment regimen (Category II) for TB in Uganda.

**Linked Research Article**

Jones-López EC, Ayakaka I, Levin J, Reilly N, Mumbowa F, et al. (2011) Effectiveness of the Standard WHO Recommended Retreatment Regimen (Category II) for Tuberculosis in Kampala, Uganda: A Prospective Cohort Study. PLoS Med 8: e427. doi:10.1371/journal.pmed.1000427

Prospective evaluation of the effectiveness of the WHO-recommended standardized retreatment regimen for tuberculosis by Edward Jones-López and colleagues reveals an unacceptable proportion of unsuccessful outcomes.

The paper by Jones-López et al. in this week's *PLoS Medicine* [1] (hereinafter “the Uganda study”) illustrates the challenge of conducting research in resource-constrained settings. At the time the study was proposed and initiated, the prevalence of multidrug resistant tuberculosis (MDR-TB) in Uganda was unknown. Further, second-line therapy for MDR-TB, available in other settings, was not available in the country. The Uganda study accordingly highlights at least two classic ethical conundrums: (1) should research be conducted in a setting if the existing standard of care for the health issue under investigation is “no treatment,” despite efficacious treatment existing elsewhere? and (2) should investigators introduce an efficacious standard of care in a setting if it would not otherwise be available?

## Is the Uganda Study Ethically Defensible? The Stance of Research Ethics Guidelines

It is worthwhile examining the ethics of the Uganda study through the lens of prominent international research ethics guidelines. Although these instruments are not binding on any setting, including Uganda, they offer helpful guidance on a range of issues that confront researchers. According to the Council for International Organizations of Medical Sciences (CIOMS) Guidelines (formally known as International Ethical Guidelines for Biomedical Research Involving Human Subjects; hereinafter “CIOMS Guideline”) [2]:

“Sponsors of research or investigators cannot, in general, be held accountable for unjust conditions where the research is conducted, but they must refrain from practices that are likely to worsen unjust conditions or contribute to new inequities…In general, the research project should leave low-resource countries or communities better off than previously or, at least, no worse off.”

On the issue of conducting research in populations and communities with limited resources, Guideline 10 of the CIOMS Guideline states that

“before undertaking research in a population or community with limited resources, the sponsor and the investigator must make every effort to ensure that:

- the research is responsive to the health needs and the priorities of the population or community in which it is to be carried out; and
- any intervention or product developed, or knowledge generated, will be made reasonably available for the benefit of that population or community.”

Guideline 10's accompanying commentary states:

“It is not sufficient simply to determine that a disease is prevalent in the population and that new or further research is needed: the ethical requirement of ‘responsiveness’ can be fulfilled only if successful interventions or other kinds of health benefit are made available to the population. This is applicable especially to research conducted in countries where governments lack the resources to make such products or benefits widely available.”

Paragraph 19 of the 2000 version of the Declaration of Helsinki [3]—which was applicable at the time the Uganda study commenced in 2003—states: “Medical research is only justified if there is a reasonable likelihood that the populations in which the research is carried out stand to benefit from the results of the research.” Paragraph 30 of the 2000 version of the Declaration of Helsinki further states: “At the conclusion of the study, every patient entered into the study should be assured of access to the best proven prophylactic, diagnostic and therapeutic methods identified by the study.”

Paragraph 30's accompanying note of clarification, which was added by the World Medical Association General Assembly in 2004 [4], states:

“The WMA hereby reaffirms its position that it is necessary during the study planning process to identify post-trial access by study participants to prophylactic, diagnostic and therapeutic procedures identified as beneficial in the study or access to other appropriate care. Post-trial access arrangements or other care must be described in the study protocol so the ethical review committee may consider such arrangements during its review.”

Paragraph 7 of the 2008 version of the Declaration of Helsinki [5]—which became applicable before the Uganda study ended—states:

“Medical research involving a disadvantaged or vulnerable population or community is only justified if the research is responsive to the health needs and priorities of this population or community and if there is a reasonable likelihood that this population or community stands to benefit from the results of the research.”

Given the above, it could be argued that the Uganda study was *unethical* if the following conditions were met:

1. The investigators initiated the study in a setting where drug-resistant TB was likely prevalent, and second-line TB therapy was not available in that setting's public sector; and
2. Notwithstanding the factors outlined in point 1, the investigators made no reasonable efforts to change this state of affairs (i.e., they left participants who required second-line therapy untreated for the entire duration of the study).

As MDR-TB was almost certainly prevalent in Uganda at the study's commencement, the Uganda study was clearly responsive to the health needs of the study setting. Moreover, the investigators made a praiseworthy good-faith attempt to initiate a second-line treatment program in Uganda, when none existed previously. To this end, they provided second-line treatment regimens to a cohort of study participants diagnosed with MDR-TB, before the study concluded. While it may be argued that the investigators could have treated more participants infected with MDR-TB with second-line drugs earlier (seven study participants with MDR-TB accessed second-line treatment later in the study through a pilot treatment program initiated by the investigators), the study is ethically defensible, if one considers that several logistic issues have to be addressed before a treatment program can be initiated.

For example, securing funding, obtaining preferentially priced second-line TB drugs, training health personnel in MDR-TB treatment, developing a directly observed therapy (DOT)-Plus program, developing laboratory capacity for drug-susceptibility testing, and establishing hospital infection control measures, are time-consuming activities and could have prevented earlier second-line therapy initiation in the study setting. Further, the Uganda study findings will, on a balance of probability, likely achieve its goal: inspire MDR-TB treatment policy reform in Uganda (and other similar settings), thereby satisfying the requirement that the local population benefit from the results of the research.

## Lessons for Others

The Uganda study holds valuable lessons for others contemplating conducting research in resource-scarce settings. While not conducting a study in a setting where no efficacious standard of care exists may, at face value and in certain instances, seem a more ethically defensible option, it could overall have more negative consequences. For instance, crucial epidemiological evidence that could demonstrate the actual state of disease prevalence in a setting, and which may be pivotal to compelling (or shaming) apathetic, obstinate, and indifferent governments to change their existing treatment policy, may end up never being yielded. Accordingly, the relevant health crisis will remain unaddressed, resulting in dire public health consequences for that setting, and in some instances, the surrounding region. The Uganda study investigators were thus justifiable in conducting the study in Uganda.

Further, as was the case in the Uganda study, the investigators' role should not be limited to merely highlighting a problem. The duty of beneficence requires investigators to assume an advocacy role. This includes making reasonable attempts to change the prevailing state of affairs (i.e., the absence of efficacious treatment in the country). To this end, investigators could follow the example of the Uganda study and provide the efficacious standard of care to study participants as soon as practically possible for the duration of the study (and, if necessary, a limited period thereafter). Moreover, they could attempt to secure an undertaking from the authorities that the state will assume the responsibility of continuing that standard of care in the study setting, post-trial, and eventually expanding its access throughout its territorial jurisdiction. Admittedly, investigators do not have the power to compel authorities to approve their study, to assume post-trial responsibilities, or to implement an efficacious standard of care beyond the study site. However, this should not stop investigators from trying to do so.

## Conclusion

The Uganda study has undoubtedly addressed an important knowledge gap in science. It will hopefully herald revisions to Uganda's TB treatment program and inspire similar reforms elsewhere. Equally significant, the study catalyzed the provision of second-line TB therapy in that country. In so doing, the study has undoubtedly left the local population better off compared to before its commencement. These two factors, alone, make the Uganda study ethically defensible.

## Abbreviations

CIOMS: Council for International Organizations of Medical Sciences
MDR-TB: multidrug resistant tuberculosis
